# PainSeeker: a head pose-invariant deep learning method for assessing rat's pain by facial expressions

**DOI:** 10.3389/fvets.2025.1619794

**Published:** 2025-10-10

**Authors:** Liu Liu, Guang Li, Dingfan Deng, Zhaoyang Li, Cheng Lu, Yuan Zong, Jinhua Yu

**Affiliations:** ^1^Department of Endodontics, The Affiliated Stomatological Hospital of Nanjing Medical University; State Key Laboratory Cultivation Base of Research, Prevention and Treatment for Oral Diseases (Nanjing Medical University), Jiangsu Province Engineering Research Center of Stomatological Translational Medicine (Nanjing Medical University), Nanjing, China; ^2^School of Biological Science and Medical Engineering, The Key Laboratory of Child Development and Learning Science of Ministry of Education, Southeast University, Nanjing, China

**Keywords:** facial expression of pain, pain assessment in rat, rat grimace scale, deep learning, attention mechanism

## Abstract

**Introduction:**

Automated assessment of pain in laboratory rats is important for both animal welfare and biomedical research. Facial expression analysis has emerged as a promising non-invasive approach for this purpose.

**Methods:**

An openly available dataset, RatsPain, was constructed, comprising images of facial expressions taken from six rats undergoing orthodontic treatment. Each image was carefully selected from pre- and post-treatment videos and annotated by eight expert pain raters using the Rat Grimace Scale (RGS). To achieve automated pain recognition, a head-pose-invariant deep learning model, PainSeeker, was developed. This model was designed to identify local facial regions strongly related to pain and to effectively learn consistently discriminative features across varying head poses.

**Results:**

Extensive experiments were conducted to evaluate PainSeeker using the RatsPain dataset. After assessing the pain conditions of each rat through facial expression analysis, all tested methods achieved good performance in terms of F-score and accuracy, significantly outperforming random guessing and providing empirical evidence for the use of facial expressions in rat pain assessment. Moreover, PainSeeker outperformed all comparison methods, with an overall F-score of 0.7731 and an accuracy rate of 74.17%, respectively.

**Discussion:**

The results demonstrate that the proposed PainSeeker model exhibits superior performance and effectiveness in automated pain assessment in rats compared with traditional machine learning and deep learning methods. This provides support for the application of facial expression analysis as a reliable tool for pain evaluation. The RatsPain dataset is freely available at https://github.com/xhzongyuan/RatsPain.

## Introduction

Rats are one of the most widely used animal species in experiments and have made significant contributions to the progress of biological and medical research (1). In various experiments conducted on rats, such as evaluating the effectiveness of painkillers through animal experiments, assessing the degree of pain in rats is crucial and indispensable (2). Facial expressions reveal emotional features such as intensity, valence, and persistence, and are correlated with neuronal activity (3). However, it is necessary to emphasize that there are certain difficulties in assessing pain in rats. Unlike humans, rats cannot express their feelings through language; therefore it is impossible to directly obtain feedback on their level of pain (4). Despite this challenge, researchers have recognized the importance of assessing pain in rats and have developed many effective methods over the past few decades (5, 6).

Currently, behavioral tests are the primary method for assessing pain in rats. Broadly, these methods can be classified into two categories: induced behavioral tests and non-induced behavioral tests (6). The Von Frey test is a well-known induction method (7, 8) and has become a common tool for evaluating pain responses in experimental animals, including rats. This test involves applying a set of calibrated filaments to specific areas of the rat (such as the paws) and gradually increasing the bending force until the filaments slightly bend the skin. By observing this slight bending, the degree of pain experienced by the rat at the corresponding force threshold could be easily determined. The Von Frey test is a widely used and relatively rapid method for assessing the pain level of rats, but it should be noted that this method requires repeated invasive stimulation of the rats, which raises concerns about the welfare of experimental animals (9).

As for non-inducing methods, wheel running analysis stands out as a non-invasive approach that can be used to assess the pain conditions of rats (10, 11). In this way, the running activities of rats can be continuously and regularly monitored. A decrease in the rats' activity levels, such as the distance and speed of running and the time spent playing on the wheel, may indicate that the rats are in a painful state. However, wheel running analysis, like many other methods for testing spontaneous behavior, requires a significant amount of time. To familiarize the rats with the movement of the rotating wheel, at least 7 days of training as a preparatory step were needed (12). Furthermore, the non-inducing behavior testing method requires specialized experimental equipment, which increases the cost of pain assessment. These drawbacks pose obstacles to the timely evaluation of pain in rats and other animals.

Recently, researchers have increasingly recognized that experimental testing methods for assessing pain in rats have numerous limitations. Therefore, they have been striving to explore whether it is possible to effectively and accurately evaluate the pain status of rats within a short period of time, without being invasive and economically feasible. This exploration benefits from research in the fields of psychology and computer vision on the relationship between individual pain expressions and facial action units, which developed a tool for assessing the degree of pain in rats called the “Rat Pain Expression Scale” (RGS) (13, 14). The RGS is simple to operate and can be completed using only facial expression images of rats. It quantifies the degree of pain in rats through four facial action units (AU) related to pain, and its convenience and effectiveness have been confirmed. In addition, compared with actual behavioral testing methods, RGS can conveniently and quickly assess the pain level of rats based solely on facial expression images, providing a completely non-invasive method that considers animal welfare.

Given that research on machine learning and deep learning methods for assessing pain in rodents (such as mice and rats) through the observation of facial expressions has been insufficient thus, this section briefly summarizes the latest progress in pain scales and related automated methods for assessing pain in laboratory rodents (mice and rats), which is highly consistent with the focus of our work. In this study, we aimed to further develop an automated approach for evaluating pain in rats based on facial expressions. This method is inspired by recent advancements in the automation of facial pain assessment in humans and other laboratory animals (15). Compared to these species, research on the automation of pain assessment in rats through facial expressions is still relatively limited. This is mainly due to the lack of well-annotated and publicly available datasets of rat data to support research in this area. To address this gap, we introduced the “RatsPain” dataset, which includes 1,138 carefully annotated and high-quality facial expression images taken from six rats that underwent orthodontic treatment. Notably, the facial expression images of the rats in our dataset exhibit diverse and challenging head postures, which is a common situation when capturing their facial videos with fixed-angle cameras. Additionally, we proposed a simple yet effective deep learning method called “PainSeeker” for automatically assessing the pain level of rats from non-frontal facial expression images. The basic idea of PainSeeker is to find stable local facial regions related to pain, ensuring that it can effectively learn features that always have the ability to distinguish pain, regardless of changes in the rat's head posture. Finally, we conducted a large number of experiments to prove the effectiveness of the proposed “PainSeeker” on the “RatsPain” dataset, thereby demonstrating that observing the facial expressions of rats to assess their pain conditions is feasible.

In summary, this study makes three major contributions.

1) We provide a detailed annotated dataset for pain assessment, named “RatsPain”. RatsPain is freely available at https://github.com/xhzongyuan/RatsPain. To our knowledge, this is the first publicly available dataset containing facial expression images of rodents in different head postures, aimed at supporting research on assessing the degree of pain in rodents through facial expressions.
2) We propose a simple yet effective deep learning method called PainSeeker to address the challenge of assessing pain in rats with diverse head poses using facial expressions.
3) We conduct extensive experiments on the RatsPain dataset, which was specifically collected to demonstrate the effectiveness and outstanding performance of the proposed PainSeeker model in assessing the pain of rats through facial expressions.

The remainder of this article is arranged as follows: the second part briefly reviews the latest progress of Grimace Scales used for pain assessment in laboratory rodents (mice and rats) and their related automated methods. The third section details the construction process of the PainSeeker dataset. The fourth section introduces the details of the proposed PainSeeker method and demonstrates its application in evaluating rat pain through facial expressions. The fifth section conducts a large number of experiments on the RatsPain dataset to evaluate the proposed PainSeeker method. Finally, in the sixth section, this article is summarized.

## Materials and methods

### Grimace scales for pain assessment in laboratory rodents

Humans can use various self-assessment scales to express the degree of their pain, such as the visual analog scale (VAS) and verbal rating scale (VRS) (16, 17). However, these scales may not be suitable for certain populations, such as infants with language impairment. Therefore, it is necessary to develop an alternative, simple, and effective method for pain assessment. Fortunately, as early as 1872, Darwin explored the possibility of using facial expressions as indicators of pain in both humans and animals. He discovered that various emotional states, including pain, could be identified through facial expressions (18).

Despite this insight, there is still no objective standard that can help people perceive and recognize facial expressions corresponding to various emotions. To fill this gap, Ekman and Friesen compiled a manual called the “Facial Action Coding System (FACS)” (19). The FACS defines a set of facial AUs that describe the movements of various facial muscles, such as raising the cheeks, and demonstrates how combinations of these AUs can encode basic facial expressions, such as happiness, fear, and anger. With the assistance of FACS, numerous studies have attempted to identify pain-related AUs and proposed various possible combinations of AUs to encode human facial expressions of pain (19–21). This led to the creation of many well-annotated datasets of human facial expressions of pain and promoted the progress of automated human pain assessment research (22, 23).

Similar to infants, laboratory rodents (mice and rats) cannot directly express their pain sensations. Moreover, the current mainstream methods used to assess the pain conditions of experimental rodents have many limitations, including long duration, high cost, and invasiveness to animals, as mentioned before. Therefore, drawing inspiration from the principles of FACS and the existing combinations of pain-related human AUs (20, 21, 24, 25), Langford et al. meticulously developed the Mouse Grimace Scale (MGS), which is highly accurate and reliable and can be used to assess the pain condition of mice through their facial expressions. In the MGS, five pain-related AUs were defined to encode pain in mice, including orbital tightening, ear position, nose bulge, whisker change, and cheek bulge. Subsequently, Sotocinal et al. (14) developed the RGS to assess the pain conditions in rats. This system was established based on the MGS system. Notably, the RGS includes only four pain-related AUs. This is because, in RGS, the Aus related to the nose and cheeks have been combined. As Sotocinal et al. discovered, in the experimental mice experiencing pain, there was a clear flattening from the nose to the cheeks, while a distinct bulge was observed in the mice. Currently, MGS and RGS have been widely used by numerous researchers to assess pain in laboratory rodents and conduct pain-related research (26).

### Automated grimace scale methods for pain assessment in laboratory rodents

While the aforementioned Grimace Scales can effectively address the limitations of current mainstream laboratory rodent pain assessment methods, it is important to note that their utilization remains labor-intensive and time-consuming. Moreover, the subjectivity introduced by raters involved in the process inevitably impacts the final annotated pain labels for rodents (27). Additionally, prolonged annotation tasks can induce rater fatigue, thereby diminishing the efficiency and accuracy of annotations based on Grimace Scales. Consequently, in recent years, several researchers have shifted their focus to the development of automated grimace scale methods for assessing pain in laboratory rodents, leveraging machine learning techniques, particularly deep learning.

One of the earliest automated Grimace Scale methods can be traced back to the work of Tuttle et al. (28), who presented an automated MGS (aMGS) to assess pain in mice. In aMGS, Inception v3 (28), a widely used convolutional neural network (CNN) structure, was used to study the pain-discriminative features from facial expression images of mice. Subsequently, Andresen et al. (29) investigated the use of various CNN architectures, including Inception v3, ResNet (30), and self-designed CNN, to automatically assess pain in mice. Pioneering work on rats began to emerge only in 2023 (31). In this study, the authors constructed an automated system for pain assessment in rats via facial expressions, consisting of a YOLOv5 model responsible for detecting the four pain-related AUs and a Vision Transformer (ViT) model responsible for discriminating pain (32).

While there has been promising progress in the research of automated Grimace Scale methods for pain assessment in laboratory rodents, they still fall short of completely replacing the original grimace scale. The main reason for this is the lack of well-annotated and publicly available animal facial expression datasets, which limits the creation of powerful machine learning and deep learning models specifically tailored for pain assessment in laboratory rodents. It is evident that in the majority of current studies on automated Grimace Scales for rodents, the training data for their models are not publicly accessible. Moreover, it is also important to note that limited by labor and time, the authors often selectively label a portion of collected facial expression samples or recruit few annotators for data annotation work, which may impact the pain label quality of established datasets.

Furthermore, it has been observed that the facial expression images of rodents in the majority of existing works were typically collected from an ideal scenario (28, 29, 31), meaning that rodents exhibit head poses with frontal or near-frontal views in these images; and hence, conventional CNN models easily achieved satisfactory performance. However, in real-world applications, mice and rats cannot consistently face the camera with a fixed view owing to their active nature or experience of pain. In this case, several pain-related AU regions may be occluded, which poses significant challenges in assessing pain in rodents based on facial expressions. Therefore, it is imperative to collect diverse samples that reflect the challenges encountered in practical scenarios to aid researchers in addressing these challenges in the future.

It is worth mentioning that the RatsPain dataset presented in this study is a well-annotated and challenging publicly available facial expression image dataset that supports the development of more practical automated methods for pain assessment in rats. We also propose a novel deep learning approach called PainSeeker to address the challenge of pain assessment in rats through non-frontal facial expressions.

## Ratspain dataset

### Animal models and video recordings of rats

**Figure 1:**
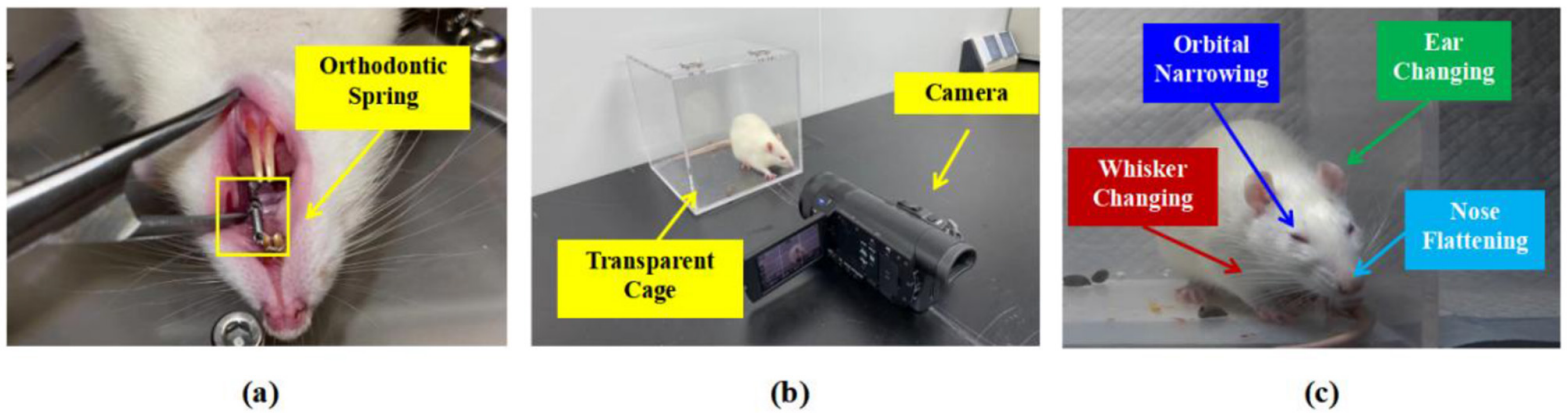
Explanation of Facial Expression Image Collection and Pain Annotation of Rats: **(a)** Rat receiving orthodontic treatment, **(b)** the environment of shooting and equipment settings, **(c)** schematic representation of labeled rats' pain levels using RGS Sotocinal et al. (14).

In this section, we provide a detailed description of the specific steps for collecting and annotating facial expression images of rodents related to pain to construct the RatsPain dataset, as described in Figure 1. Firstly, 6 healthy male Sprague-Dawley (SD) rodents aged ~8 weeks underwent orthodontic treatment to cause pain, as shown in Figure 1a. The force applied by the orthodontic spring used in the treatment was 0.8 Newtons. It should be noted that each rodent was placed in a crystalline cage individually and was equipped with a front-facing camera, as shown in Figure 1b. Additionally, for each rodent, the video camera captured 1-h videos before and after the orthodontic treatment. Specifically, since the pain experienced by the mice typically peaks within ~24–48 h after orthodontic treatment (33), the post-operative video was taken 1 day after the treatment, while the pre-operative video was captured 1 h before the treatment. The animal experiments in this paper have been approved by the Institutional Animal Use Committee of Nanjing Medical University, and the Ethics code number was IACUC-2406023.

### Selection of images of facial expression in rat

After the recording was completed, we established strict exclusion and inclusion criteria to standardize the subsequent manual screening process for rats with high-quality facial expressions related to pain. Specifically, the facial expression images of rats that meet the criteria and can be included in the dataset should clearly show the four key pain-related facial AUs: ears, nose, whiskers, and eyes, as shown in Figure 1c. It is worth noting that partial occlusion of one eye, whiskers, or both is acceptable because such omission will not have a significant impact on the assessment of the rats' pain level based on the corresponding facial expression images. However, during the screening process, images depicting the rats in the process of grooming, standing, or sleeping are excluded because these images cannot accurately assess the pain level through the observation of facial expressions.

To carry out this screening work, five out of the seven authors were responsible for manually collecting facial expression images from the original videos of mice, while the other two authors were responsible for reviewing and confirming whether the collected facial expression images met the aforementioned standards. It should be noted that to ensure the diversity of the samples, at most five images per minute could be extracted from the videos for unified collection. Eventually, we obtained a total of 1,295 high-quality facial expression images related to pain from six mice.

### Explanation of pain levels of rats

To acquire highly reliable image-level pain annotations for the selected facial images of the experimental mice, we additionally recruited eight well-trained undergraduate/graduate students as pain assessors. Each student received detailed training on RGS and was able to proficiently distinguish different pain rating levels: 0 point represents no pain, 1 point represents moderate pain, and 2 points represents severe pain, based on one of four facial AUs in rats, as depicted in Figure 1c.

Subsequently, they performed a two-stage labeling scheme for the facial expression images of these selected laboratory rats. Specifically, five students were assigned to the first stage, tasked with assigning the three pain scores to each facial expression image based on four pain-related facial AUs, respectively, thereby serving as the pain labels for the AU levels. It should be noted that due to potential issues with image quality, such as non-orthogonal head postures and changes in lighting, some students inevitably encountered difficulties in determining appropriate pain scores for certain facial action units of the laboratory mice (such as whiskers). To address this problem, we allowed the use of “uncertain” AU-level pain labels to cover such situations.

During the second period, the left three students went on giving AU-level pain scores in a low confidence manner for facial expression images containing AUs. An AU with low confidence is defined here as the AU for which fewer than four out of five students assigned the same score during the first stage. Conversely, the remaining AUs can be considered those with high confidence, and their consistent pain scores given by four or five students can serve as the AU-level pain labels. With the assistance of the additional three students, we then checked over the distribution of scores on the all of the eight pain scores of the AUs in a low confidence manner. Besides, in this stage, only AUs that received no less than five same scores were accepted.

Based on the two-stage labeling scheme, three or four high-confidence AU-level pain scores for 1,138 of 1,295 images are eventually attained. And the rounded mean of the AU-level pain scores was hence applied as the image-level pain label for each expression image. In addition, as for the left 157 images, their image-level pain labels weren't appointed because of massive low-confident AUs.

### A glimpse at the ratspain dataset

To offer readers an overview of the RatsPain dataset, we recorded statistics in Table 1. It is clear that the number of facial expression images labeled as “Severe Pain” in our RatsPain dataset is only six, which is significantly fewer than those labeled as “Moderate Pain” (591 images). We consider this sample distribution is reasonable, as orthodontic treatment typically does not induce consistently high-intensity pain. This assertion can be supported by the study of Liao et al. (34), in which the RGS scores of rats undergoing orthodontic-induced pain generally remain at one or less within 1 day.

**Table 1:** The sample statistics of the ratspain dataset.

**Sample subset**	**Pain label**	**# Expression images**	
	No	541	
High Confidence	Moderate	591	1,138
	Severe	6	
Low Confidence	N/A	157	
Total		1,295	

Moreover, we present the sample statistical information for the high-confidence subset of the RatsPain dataset in Table 2. It is revealed a prevalent class-imbalanced problem in Rats#1, #3, #4, #5, and #6. Specifically, the number of samples labeled as “Pain” (“Moderate” and “Severe”) is significantly larger than that of samples labeled as “No Pain” for Rats#1, #5, and #6, while the opposite situation exists in Rats#3 and #4.

**Table 2:** The sample statistical information of the high confidence subset in the ratspain dataset.

**Sample subset**	**Pain label**	**# Expression images**	
Rat#1	No	56	221
	Moderate	163	
	Severe	2	
Rat#2	No	102	210
	Moderate	108	
	Severe	0	
Rat#3	No	148	198
	Moderate	50	
	Severe	0	
Rat#4	No	138	230
	Moderate	92	
	Severe	0	
Rat#5	No	63	182
	Moderate	116	
	Severe	3	
Rat#6	No	34	97
	Moderate	62	
	Severe	1	
Total			1,138

**Figure 2:**
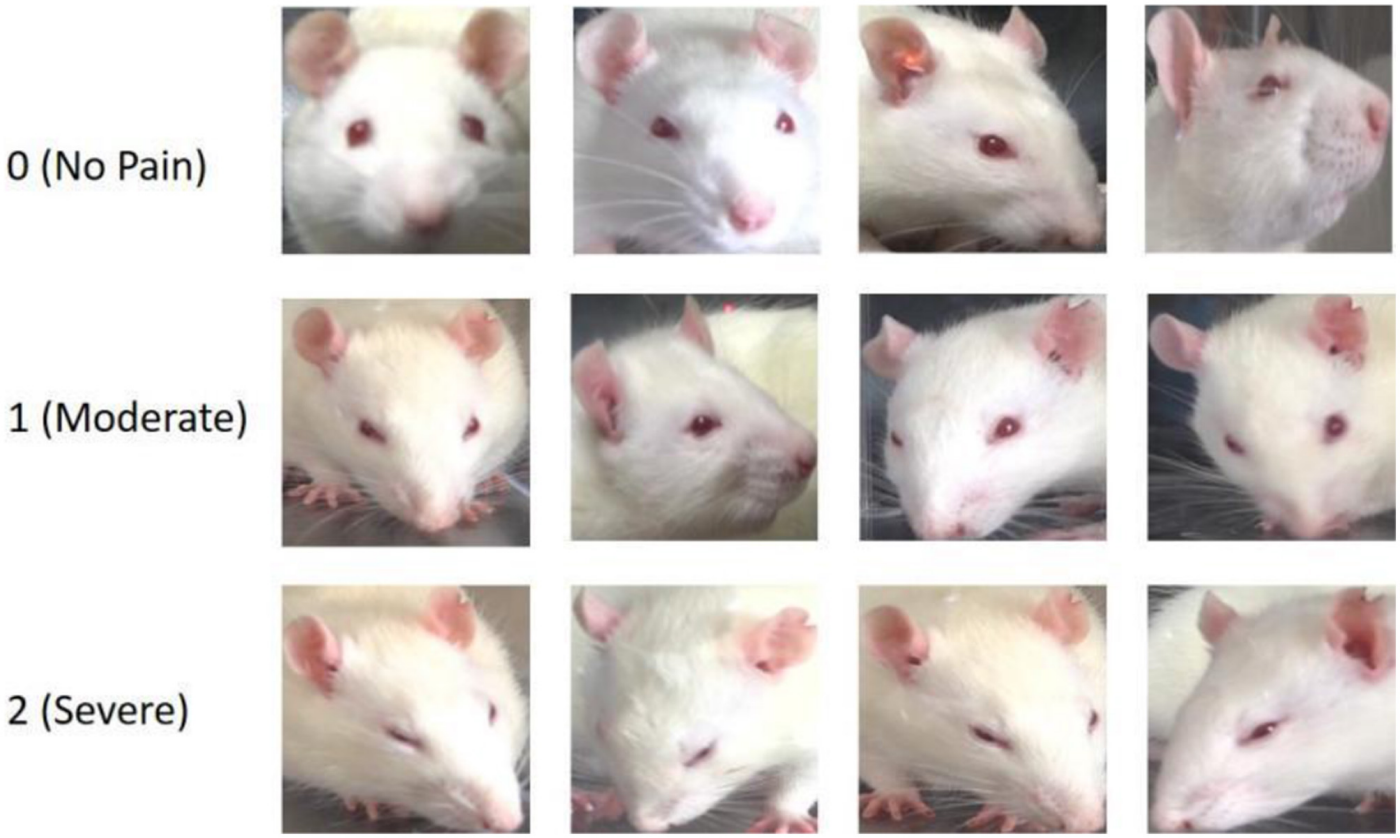
Facial images of rats expressing different pain levels from the ratspain dataset.

In addition, we present a range of facial expression samples extracted from our RatsPain dataset, illustrating various degrees of pain, as depicted in Figure 2. The figure clearly demonstrates noticeable alterations in the majority of the four pain-related AUs evident in rats' facial expressions labeled as both “Moderate Pain” and “Severe Pain”, in comparison to those labeled as “No Pain”. Moreover, it is worth noting that the head poses of rats in our RatsPain dataset encompass a variety of challenging perspectives commonly encountered in real-world scenarios.

## Painseeker for evaluating pain of rats by non-frontal facial expressions

### Basic ideas and preliminary assumptions

**Figure 3:**
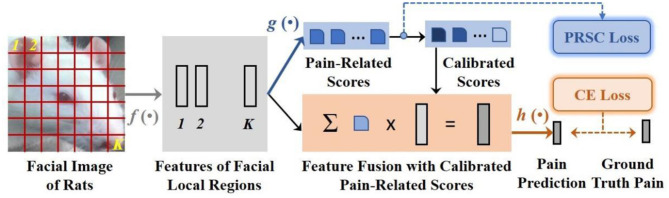
Integral structure of PainSeeker for evaluating rats' pain by facial expressions.

The basic concept of the PainSeeker model originated from our observations of rats placed in crystal cages. We discovered that owing to rat's active nature and the discomfort caused by orthodontic treatment, they often showed irregular movements. These movements always led to continuous changes in the head perspective and head posture in the facial expression images captured by the fixed-position camera. As mentioned earlier, this resulted in the occlusion of several action units related to pain, making it difficult to identify pain characteristics from the mice's facial expressions. To address this challenge, we proposed a new deep learning method named PainSeeker, as illustrated in Figure 3. The primary purpose of the PainSeeker model is to find the facial local regions highly related to pain and enhance their robustness to head pose variance, thereby facilitating more effective learning of both postural invariance and pain differentiation features from rats' facial expression images.

In the next section, we will describe the PainSeeker in detail. Firstly, we introduce a training set comprising of *N* images of the rats' facial expression illustrated as $\left\{X_{1},\cdots \;,X_{N}\right\}$. Each image, $X_{i}\in {\mathbb{R}}^{d\times d\times 3}$, indicates a colored picture with three channels and pixels *d* × *d*. The matching label is illustrated as ${\text{y}}_{i}\in {\mathbb{R}}^{c\times 1}$, which is a one-hot vector created on the basis of the ground truth pain levels scoping from 1 to *c*. As described in Figure 3, at first, an expression image of one rat is passed through a suit of convolutional layers, leading to a group of original features relevant to *K* = *M* × *M* local regions of face. The characteristics can be delineated as:


(1) $$\begin{array}{l} \left[{\text{x}}_{i,1},\cdots \;,{\text{x}}_{i,K}\right]=Reshape\left(f\left(X_{i}\right),\left[K,d_{x}\right]\right), \end{array}$$

where *Reshape* (·) refers the process of reshaping the tensor $f\left(X_{i}\right)\in {\mathbb{R}}^{M\times M\times d_{x}}$ into a *d*_*x*_−*by*−*K* matrix, *f*(·) stands for the operation conducted applying a set of convolutional layers, *K* denotes the size of characteristic patterns in the last convolutional layer in *f*(·). In addition, *d*_*x*_ represents the number of feature maps, respectively.

### Measuring contributions of rats' facial local regions related to pain

In the place of directly flattening these features related to the facial local regions, as is the case with the widely used structure of CNNs, e.g., VGG (40), our PainSeeker model incorporates the attention mechanism (41) to appoint a Fully-connected (FC) layer which measures out the contribution scores correlated with pain of features relevant to different local regions of face. And it can be formulated as:


(2) $$\begin{array}{l} \beta_{i,j}=\frac{\sigma \left(g\left({\text{x}}_{i,j}\right)\right)}{\overset{K}{\underset{j=1}{\sum }}\sigma \left(g\left({\text{x}}_{i,j}\right)\right)}, \end{array}$$

where *j* = {1, ⋯ , *K*}, *g*(·) illustrates the operation conducted by the FC layer, σ(·) denotes the sigmoid function, respectively.

By resorting to these contribution scores related to pain, we could fuse the original features of facial local regions and then estimate the pain level of the rats' expression image according to its feature fusion, which can be illustrated as:


(3) $$\begin{array}{l} y_{i}^{p}=Softmax\left(h\left(\overset{K}{\underset{j=1}{\sum }}\beta_{i,j}x_{i,j}\right)\right) \end{array}$$

where *h*(·) and softmax (·) represent the procedures conducted by a FC layer and softmax function. In order to train the PainSeeker model, we apply the cross-entropy (CE) loss to set up the relationship between the pain label anticipated by PainSeeker and the corresponding ground truth. The CE Loss are illustrated as:


(4) $$\begin{array}{l} L_{CE}=J\left({\text{y}}_{i},{\text{y}}_{i}^{p}\right), \end{array}$$

Where J(·) is the CE function.

### Highlighting highly-contributive rats' pain-related facial local regions

It is crucial to notice that minimizing the CE loss in Equation (4) enables us to get a set of β_*i, j*_ values scoping from 0 to 1. Large β_*i, j*_ values indicate strong associations of the corresponding facial local regions with pain in rats, thus contributing highly to learning distinguishing features for anticipating the rats' pain level. But the association of these highly-contributive facial local regions may reduce due to partial occlusion caused by deviations in head poses of rats in a frontal perspective. As a consequence, the values of these regions, ideally large, may show a narrower disparity in comparison with the regions that are less-contributive in practical scenarios.

In terms of this issue, we came up with a new regularization term named pain-related score calibration (PRSC) for the PainSeeker model, stemming from the widely-applied triplet loss (42). And the PRSC loss function is shown as follows:


(5) $$\begin{array}{l} L_{PRSC}=\frac{1}{K_{h}}\sum_{j=1}^{K_{h}}\max\left\{0,\delta -\left(\overset{h}{\underset{i,j}{\beta }}-{\bar{\beta }}_{i}^{r}\right)\right\}, \end{array}$$

In Equation (5), δ denotes the preset margin value, $\beta_{i,j}^{h}$ represents the *j*^th^ element among the highest K_*h*_ scores that are highly related to pain, ${\bar{\beta }}_{i}^{r}$ denotes the average value among the *K*_*r*_ = *K*−*K*_*h*_ resting scores relevant to the local regions of face that are less-contributive. The concurrent minimization of the PRSC loss and the CE loss leads to a wider disparity between the scores that are highly related to pain and the other scores. This procedure highlights the features studied from local regions of faces that are highly related to pain while restraining these features that are less-contributive in the original fused features shown in Equation (3). Through this calibration, the feature fusion facilitated by the PainSeeker model becomes more robust to variations in head pose while retaining its discriminative capability for pain assessment.

### Optimization problem for painseeker

Associating the loss functions exhibited in Equations (4) and (5), and summing overall these *N* training samples, we can obtain the total loss function for the suggested PainSeeker model. The optimiation problem for training PainSeeker model can be expressed as follows:


(6) $$\begin{array}{r} \min_{\theta_{f},\theta_{g},\theta_{h}}\frac{1}{N}\sum_{i=1}^{N}[J\left(y_{i},y_{i}^{p}\right)\\ +\frac{\lambda }{K_{h}}\sum_{j=1}^{K_{h}}\max\{0,\delta -\left(\beta_{i,j}^{h}-{\bar{\beta }}_{i}^{r}\right)\}],\; \end{array}$$

where θ_*f*_, θ_*g*_, θ_*h*_ stands for the parameters related to the functions conducted employing the layers *f* , *g*, and *h* in PainSeeker, λ is the trade-off parameter to keep the balance between PRSC and CE losses, respectively.

### Pain label prediction using the trained Painseeker model

The optimization problem presented in Equation (6) can be efficiently solved using widely-used optimizers such as SGD and Adam. Once the solution is obtained, the pain label of a testing rat's facial expression image can be predicted using Equation (3). The input for this equation corresponds to the set of original features derived from the test image, obtained by applying the reshape operation described in Equation (1).

## Experiments

### Experimental protocol

During this process, we carry on extensive pain assessment experiments on the high-confidence sample set of the RatsPain dataset to assess the PainSeeker model. Because of the finite number of samples labeled with severe pain, we decide to unite both moderate and severe samples into the “Pain” category, enabling us to take binary classification tasks. Then we applied the leave-one-rat-out (LORO) protocol for valuation, implementing *S* folds of experiments, where *S* acts on behalf of the quantity of rats included in the dataset. As for every fold of the research, we will utilize the expression images of one rat to be the testing set, in the meantime, the expression images of the left rats are applied as the training set at the same time.

The performance criterion used are the *F1-score* and *Accuracy*, which will worked out by the following formulas:


(7) $$\begin{array}{l} F1-score=\frac{2TP}{2TP+FP+FN}, \end{array}$$

(8) $$\begin{array}{l} Accuracy=\frac{TN+TP}{TP+FP+TN+FN}\times 100.\; \end{array}$$

In this formulas, TP, TN, FP, and FN stands for the numbers of expression images of rats across all the folds properly anticipated as pain, improperly anticipated as pain, wrongly anticipated as no pain, and anticipated as no pain labels in a correct manner, respectively. In time of the researches, we cropped artificially every rat's expression image from the raw image, including the four key pain-related AUs, as exhibited in Figure 2. Then these clipped images were changed the size to 224 × 224 pixels.

### Comparison methods and implementation detail

In our experiments, we employ the convolutional layers of ResNet-18 to extract original features from the local regions of face for PainSeeker (30). The trade-off parameter λ, margin value δ, and the number of local regions of face that are highly related to pain. *K*_*h*_ in PainSeeker are fixed at 0.1, 0.2, and 5, respectively. In the training stage, we set the batch size to 64, employ the Adam optimizer, the learning rate and weight decay are set to 1e^−4^ and 0.01. Moreover, we employ a sample augmentation strategy. Specifically, we first resize each rat facial image to 256 × 256 pixels and then randomly crop a sub-region with 224 × 224 pixels to generate more diverse training rat facial images. The horizontal flipping operation is also employed to double the training samples. To appraise the performance of PainSeeker in the light of the challenge of assessing rats' pain by non-frontal facial expressions, we compare it with several deep learning and machine learning methods. The deep learning method chosen for comparison is ResNet-18. We maintain identical settings for batch size, optimizer, learning rate, and sample augmentation during training, as those used for PainSeeker.

As for the machine learning methods, we employ an association of Local Binary Pattern (LBP) and Support Vector Machine (SVM) (35, 36). As for this approach, LBP is utilized to extract features describing the facial expression images of rats, while SVM handles the subsequent pain classification task. For LBP feature extraction, following previous works in human facial expression recognition (37, 38), we first divide the rat facial image into a set of facial local regions with a fixed-size spatial grid, e.g., 4 × 4. LBP features are then extracted from these facial local regions and concatenated into a feature vector to describe the rat facial image. Note that three types of spatial grids, including 4 × 4, 8 × 8, and 16 × 16, are used in the experiments. Moreover, LBP has two important parameters to be set: the number of neighboring pixels *R* and the radius *P*. We set *R* = 1, 3 and *P* = 8, resulting in six combinations of LBP and SVM, denoted as LBP_R1P8_ (4 × 4), LBP_R1P8_ (8 × 8), LBP_R1P8_ (16 × 16), LBP_R3P8_ (4 × 4), LBP_R3P8_ (8 × 8), and LBP_R1P8_ (16 × 16). Additionally, the kernel function chosen for SVM is the linear kernel, defined as *k*(*x, y*) = *x*^*T*^*y*, where *x* and *y* are input vectors. The penalty coefficient for SVM is fixed at *C* = 1.

## Results and discussions

The experimental results of these methods were shown in Table 3. Several interesting conclusions can be drawn from them.

**Table 3:** Comparison of f1-score and accuracy of evaluating pain of rats by expressions under the loro protocol.

**Method**	**Rat#1**	**Rat#2**	**Rat#3**	**Rat#4**	**Rat#5**	**Rat#6**	**LORO**
LBP_R1P8_ (4 × 4)	0.7862/71.95	0.7292/64.29	0.5399/62.12	0.6291/65.65	0.6923/64.84	0.6087/62.89	0.6854/65.64
LBP_R1P8_ (8 × 8)	0.7835/71.49	0.7396/67.14	0.4906/59.09	0.6184/65.65	0.6300/59.34	0.5493/57.73	0.6853/64.24
LBP_R1P8_ (16 × 16)	0.7703/69.23	0.7068/65.24	0.4648/61.62	0.5326/62.61	0.5930/55.49	0.7000/69.07	0.6462/63.62
LBP_R3P8_ (4 × 4)	0.8014/74.66	0.7341/66.19	0.5223/62.12	0.5842/63.48	0.7184/68.13	0.7736/75.26	0.6984/67.66
LBP_R3P8_ (8 × 8)	0.7286/66.97	0.7315/67.14	0.4247/57.58	0.6193/67.39	0.7264/68.13	0.6800/67.01	0.6689/65.64
LBP_R3P8_ (16 × 16)	0.7416/68.78	0.7160/65.24	0.4460/61.11	0.5510/61.74	0.5333/50.00	0.6346/60.82	0.6235/61.69
ResNet-18	0.8802/80.54	0.7004/66.19	0.4274/66.16	0.6240/59.13	0.7905/65.93	0.8029/72.16	0.7393/68.01
PainSeeker w/o PRSC	0.8516/75.57	0.7045/62.86	0.5263/77.27	0.5689/57.83	0.7386/65.38	0.7931/75.26	0.7234/68.28
PainSeeker (Ours)	0.8944/84.62	0.7490/70.00	0.5586/75.25	0.6847/69.57	0.8000/71.43	0.8000/73.20	0.7731/74.17

First, after evaluating the pain conditions of each rat through facial expression assessment, all methods demonstrated excellent performance in terms of *F1-score* and *Accuracy*. In PainSeeker, the *Accuracy, Recall rate* and *F1-score* are calculated based on the confusion matrix and are used to evaluate the classification performance of the model for animal pain expressions. The results were significantly better than random guessing, which provided strong research-based evidence for the view that facial expressions can be used to assess the pain levels of rats. Moreover, it should be noticed that our PainSeeker model outperformed all the comparison methods, achieving remarkable comprehensive *F1-score* and *Accuracy* of 0.7731 and 74.17%, respectively. It proved that the proposed PainSeeker model has superior performance and effectiveness in handling this increasingly growing and highly significant issue compared to traditional machine learning and deep learning methods. In other words, for the challenges involved in assessing the pain status of rats through facial expressions, (especially the non-positive perspective emphasized in this study), adopting targeted methods such as the PainSeeker model is more effective than directly applying existing traditional machine learning and deep learning techniques.

Second, we present the *F1-score* and *Accuracy* achieved by every rat across all methods shown in Table 3. The results figure out an intriguing trend, with consistently significant performance differences exists Rats#3 and #4 compared to the remaining rats across nearly all methods. This finding inspires us to take the potential of a class-imbalanced problem in pain assessment via facial expressions into account when employing samples from these rats as the testing set. It is known that an extremely class-imbalanced problem existing in the training set can affect the effectiveness of machine learning approaches (39). To investigate this aspect, we present the training and testing sample numbers across all folds of our experiments in Table 4. Furthermore, it is evident that the gap between “No Pain” and “Pain” training sample numbers exceeds 100 (the second most severe) for Rat#4 and surpasses 150 for Rat#3 (most severe). Consequently, all methods exhibit inferior performance when using Rat#3 as the testing subject compared to Rat#4, as shown in Table 4. Therefore, further investigation is warranted to explore this aspect and mitigate this interference in the development of both deep learning and machine learning methods for evaluating rats' pain through facial expressions.

**Table 4:** The sample statistical information of the training set in each fold of experiments on ratspain dataset under the Loro protocol.

**Testing Rat**	**# Training Rat Facial Images**	
	**“No Pain”**	**“Pain”**
Rat#1	485	432
Rat#2	439	489
Rat#3	393	547
Rat#4	403	505
Rat#5	478	478
Rat#6	507	534

### Further exploration of the Painseeker model

In this section, we delve deeper into the proposed PainSeeker model to provide readers with a more comprehensive understanding of its advantages in addressing the challenge of assessing pain in rats from non-frontal facial expressions. Specifically, we aim to evaluate the efficacy of the meticulously designed PRSC loss (a new regularization term designed to help the PainSeeker model identify pain discrimination and head posture invariant features from the rats' expressions). To this end, we exclude the PRSC term from the overall loss function of the initial PainSeeker model, leading to a simplified version referred to as PainSeeker w/o PRSC. Subsequently, we conduct LORO investigations using the parameter configurations as those employed for the original PainSeeker. Table 3 presents a summary of all the results.

As depicted in the table, PainSeeker w/o PRSC achieves a *F1-score* of 0.7234 and an *Accuracy* of 68.28% on the RatsPain dataset. This is significantly lower than that of the original PainSeeker model, which had an *F1-score* of 0.7731 and an *Accuracy* of 74.17% on this dataset. This comparative analysis clearly demonstrates that the introduction of the PRSC loss effectively improved the performance of PainSeeker in evaluating the pain of rats, especially when their head postures were presented in diverse non-positive perspectives through facial expressions.

**Figure 4:**
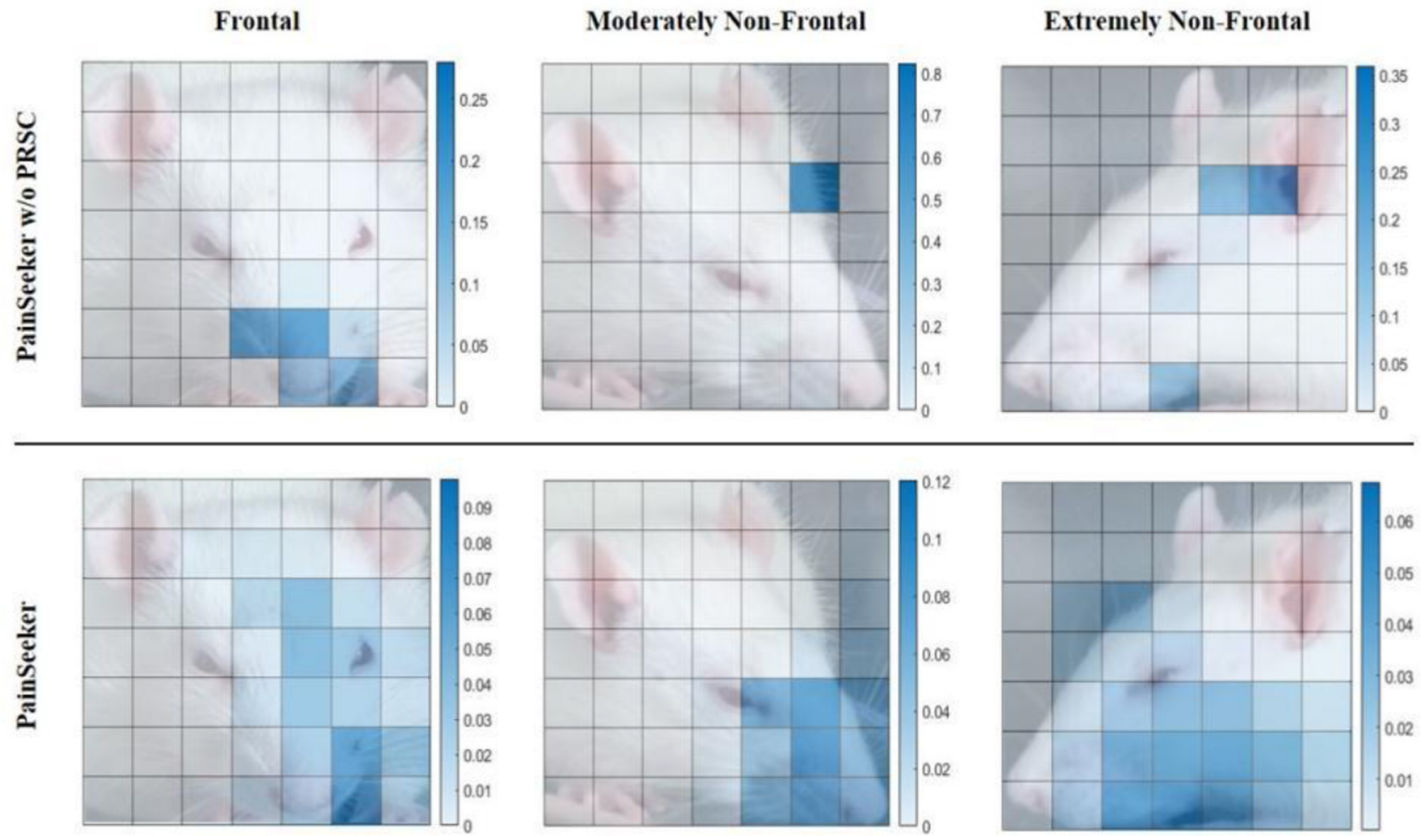
Results of pain-related contribution scores learned by PainSeeker without PRSC and painseeker for rat facial expression images with frontal, moderately non-frontal, and extremely non-frontal views, respectively.

Furthermore, we aim to clarify through experiments why, in the PainSeeker model with PRSC added, features related to pain can be learned from the facial expressions of mice, regardless of the differences in the head postures of the mice. Specifically, we selected three labeled “Pain” rat facial expression images from the first round of tests of the previous LORO experiment. The head postures of these images corresponded to frontal, moderately non-frontal, and extremely non-frontal head postures. Then, we generated heat maps for them based on the contribution scores β_*i, j*_ related to pain that were learned jointly by the PainSeeker w/o PRSC and the original PainSeeker models. The experimental results are shown in Figure 4. The top row corresponds to the results of PainSeeker w/o PRSC, while the bottom row corresponds to the results of the original PainSeeker.

From the visualization results, it is evident that both models can effectively focus attention on the key facial local areas in the facial expression images of rodents with positive head postures, such as the nose, whiskers, and cheeks, which are closely related to pain as defined in the RGS. The contribution values related to pain in these areas are significantly higher than those in less relevant facial areas. However, for the facial expression samples of rodents with non-positive head postures, PainSeeker w/o PRSC often fails to focus on these key facial local areas. Moreover, there is also a significant difference in these focus areas in terms of numerical values, being larger compared to other areas. On the contrary, after introducing PRCS in the PainSeeker model, it will prompt itself to focus more on the truly pain-related facial local areas, thereby obtaining higher values for the contribution scores related to pain. This situation holds true regardless of whether the head posture is moderately or extremely non-positive, thus alleviating the problem of insufficient utilization of facial local areas related to pain.

DeepLabCut is a universal motion tracking tool that can mark and analyze the 2D/3D movement trajectories of any part of an animal's body (such as the face, limbs, tail). Its functions are more extensive and are applicable to multiple fields such as behavior studies and neuroscience. In terms of analyzing facial expressions, PainSeeker has the following advantages over DeepLabCut: First, PainSeeker is specifically designed for the analysis of facial expressions related to pain, focusing on identifying changes in facial expressions caused by pain in animals, such as frowning, changes in ear position. It can directly quantify the intensity of pain and is suitable for scenarios such as postoperative pain monitoring and pain assessment of experimental animals. However, DeepLabCut, as a general motion tracking tool, requires users to define key points themselves, and its specificity in pain expression analysis is relatively weak. Second, PainSeeker has preset models for pain analysis, and the operation is simple, making it suitable for non-technical users to quickly get started. However, DeepLabCut requires users to have a certain programming foundation and manual annotation ability, and the learning curve is relatively steep. PainSeeker may enhance the accuracy of pain assessment by integrating multimodal data such as sounds and physiological indicators, while DeepLabCut mainly analyzes based on video data and is relatively limited in data fusion. Third, PainSeeker may enhance the accuracy of pain assessment by integrating multimodal data such as sounds and physiological indicators, while DeepLabCut mainly analyzes based on video data and is relatively limited in data fusion. In addition, PainSeeker directly provides pain scores or classification results, making it easy for users to quickly interpret the data. On the other hand, DeepLabCut outputs key point coordinates or movement trajectories, which require users to conduct further analysis and have higher requirements for data processing capabilities.

### Hyper-parameter sensitivity analysis for the painseeker model

From Equation (6), it is evident that the performance of the PainSeeker model relies on three key hyper-parameters necessitating configuration for rats' pain assessment effectively by observing facial expressions: the trade-off parameter λ, margin value δ, and the number of local regions of face that are highly related to pain *K*_*h*_. This naturally leads to a question: How do these changes in the hyperparameters affect the performance of PainSeeker? To investigate this, we conducted a large number of hyperparameter sensitivity analysis experiments using the RatsPain dataset.

**Figure 5:**
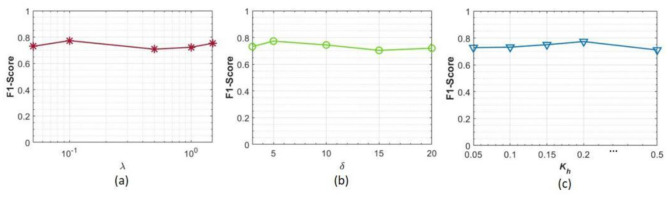
Results of sensitivity analysis experiments for hyper-parameters in the proposed PainSeeker model, where **(a–c)** correspond to the outcomes of the trade-off parameter λ, the margin value δ, and the number of highly pain-related facial local regions *K*_*h*_, respectively.

Specifically, we maintain one of the three hyper-parameters at values employed in Section V-B (λ = 0.1, δ = 0.2, and *K*_*h*_= 5), while allowing the other two to vary within predefined parameter intervals: λε [0.05, 0.1, 0.5, 1, 1.5], δε [0.05, 0.1, 0.15, 0.2, 0.5], and *K*_*h*_ε (3, 5, 10, 15, 20). The experimental setup follows the LORO protocol, with batch size, optimizer, learning rate, and weight decay consistent with previous experiments. The results are presented in Figure 5.

It is easy to see from this chart that the performance of our PainSeeker model shows only minor differences in response when any one of the three trade-off parameters changes. This indicates that the PainSeeker model is relatively insensitive to fluctuations in its hyperparameters when assessing the pain situation of rats through facial expressions. Therefore, in practical applications, it may not be necessary to conduct complex hyperparameter selection for the PainSeeker model.

## Limitations

However, we acknowledge that there are still many shortcomings in our work, which need to be further improved in the future. Firstly, the RatsPain database only covers one type of pain stimulus for rats, namely orthodontic treatment. It is necessary to study whether our PainSeeker can effectively process facial expression samples of rats that have undergone other pain stimulation methods. Additionally, we also question whether our PainSeeker model is applicable to rats from different strains, genders, and age groups. Therefore, it is necessary to further collect and analyze facial expression samples from different rats. Therefore, we will continue to expand the RatsPain database and evaluate the effectiveness of the PainSeeker method in assessing rat pain in a more natural environment in the future. At the same time, we will also strive to design more effective deep learning methods to address the challenges of assessing rat pain in a more natural environment.

## Conclusion

Our study demonstrates the effectiveness of the proposed “PainSeeker” model, especially its advantages in addressing head posture variations, and provides experimental evidence for the possibility of pain assessment through facial expression observation.

## Data Availability

The original contributions presented in the study are included in the article/supplementary material, further inquiries can be directed to the corresponding authors.
